# The Incidence and Classification of the Severity of Heterotopic Ossification Around Femoral Endoprostheses

**DOI:** 10.5435/JAAOSGlobal-D-25-00052

**Published:** 2025-12-09

**Authors:** Drew D. Moore, Jeffrey W. Lamping, Kevin W. Park

**Affiliations:** From the Department of Orthopaedic Surgery, William Beaumont University Hospital, Corewell Health, Royal Oak, MI (Dr. Moore, Dr. Lamping, and Dr. Park), and the Oakland University William Beaumont School of Medicine, Rochester, MI (Dr. Moore).

## Abstract

**Introduction:**

Although several studies observe and report the incidence of heterotopic ossification (HO) following total hip arthroplasty, to our knowledge, no study has characterized HO around femoral endoprostheses for reconstruction in cases of tumor resection or massive bone loss.

**Methods:**

A grading system for HO around a femoral endoprosthesis was developed based on the Brooker Classification System. We then retrospectively reviewed all patients who underwent primary femoral endoprosthetic reconstruction (proximal, distal, or total) between 2010 and 2017 with minimum 6-month follow-up at a single institution. Radiographs from the most recent follow-up were used to classify the pattern of HO using the defined classification system. Intra- and interrater reliabilities of the classification system were determined. Electronic medical records were reviewed for patient demographics and further information on treatment.

**Results:**

Forty-one femoral endoprostheses in 41 patients were included (22 proximal, 15 distal, and four total femur arthroplasties). Mean age at surgery was 53 ± 23 years (range 9 to 91 years), and 22 were female (54%). Inter- and intrarater agreement for HO classification was 87% (κ = 0.82) and 91% (κ = 0.88), respectively. In total, an 83% incidence of HO was found after femoral endoprosthetic arthroplasty. All proximal femur arthroplasties and 66.7% of distal femoral arthroplasties exhibited some degree of HO. Patients with HO were markedly older than those without (57 ± 21 vs. 30 ± 19; *P* = 0.003).

**Discussion:**

The proposed classification system has high reproducibility and agreement. Our data suggest that HO is very common after femoral endoprosthetic reconstruction and is more predominant around the hip compared with the knee. Most HO was noted around the diaphyseal stem of the prosthesis and away from the joint.

Heterotopic ossification (HO) is the formation of non-skeletal, mature, lamellar bone in soft tissue, including tendon and muscle.^1,2^ HO is a common occurrence after major orthopaedic surgery.^1^ It occurs most frequently after hip surgery, including total hip arthroplasty (THA), and although less common, can also occur after total knee arthroplasty (TKA).^1,3^ Its clinical impact on the patient is variable, ranging from being entirely asymptomatic to causing decreased joint range of motion and even joint ankylosis in severe cases.^1^ The clinical severity is often dependent on the extent of bone growth. HO in the setting of THA has been well described. Several studies have reported on the incidence of HO after THA, ranging from 5.2% to 87% in overall occurrence, with 0.5% to 12.3% being clinically notable.^4-10^ Risk factors, prevention, and outcomes of HO for THA have also been widely reported.^1,4,9-11^

HO can also present after femoral endoprosthetic reconstruction; however, to our knowledge, the incidence has not yet been described in the literature. An endoprosthesis is necessary in cases that require extensive bone resection; it is often performed for reconstruction and stabilization of massive bone defects such as following resection of primary bone sarcomas.^12^ Arthroplasty with a femoral endoprosthesis is a more invasive procedure than a THA or TKA. Given the differences in surgical indication and invasiveness, we felt that the incidence and classification of HO after total femoral arthroplasty should not be directly extrapolated from the THA and TKA literature.

Several classification schemes have been proposed for describing the degree of HO after THA, but the most widely used is the Brooker Classification System, a scale that ranges from I to IV depending on the amount of ectopic bone growth.^13^ Previous studies have modified the Brooker Classification System to describe HO for causes other than THA.^14^ The purpose of this study was to develop a novel classification system, based on the Brooker Classification, to describe patterns of HO around femoral endoprostheses and to use that classification system to determine the incidence of HO after femoral endoprosthetic reconstruction. In addition, we aimed to use these data to find possible risk factors for developing HO in this setting. We hypothesized that there would be a high incidence of HO based on our clinical observations.

## Methods

With approval from our institutional review board, we retrospectively reviewed all patients undergoing primary femoral endoprosthetic reconstruction between 2010 and 2017 at a single, tertiary care, cancer institute by one of two fellowship-trained dedicated orthopaedic oncologists, in practice >5 and >20 years, respectively, at the time of study. Proximal, distal, and total femoral endoprostheses were included. Surgical technique included a lateral approach for all proximal and total femurs. Distal femoral endoprosthesis technique was variable based on prior approaches for revision case bone loss, or location and extent of tumor excision for oncologic cases. Of 15 distal femur endoprostheses, eight were through lateral approach, three midline medial peripatellar, and four medial. Patients with <6 months of follow-up were excluded. Previous reports have found that HO is generally mature and recognizable by 6 months postoperative.^13,15^ Electronic medical records were reviewed for patient demographics, type of implant, indication for procedure, and further information on treatment, including whether patients were treated with radiation therapy postoperatively. Of note, no patients received dedicated HO prophylaxis in the form of prophylactic medication or radiation.

**Figure 1:**
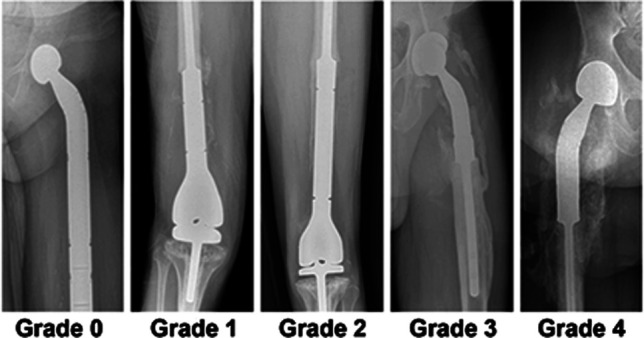
Radiographs demonstrating examples of grade 1 through grade 5 for the classification of heterotopic ossification around a femoral endoprostheses.

A grading system for HO around a femoral endoprosthesis was developed based on the Brooker Classification System (Table 1). A score of 0 = no HO, 1 = bone island within soft tissue and >1 cm from the joint articulation or opposing bone surface, 2 = bone spur from the bone-implant junction or diaphysis and >1 cm from the joint articulation or opposing bone surface, 3 = bone spur or island <1 cm from the joint articulation or opposing bone surface, and 4 = apparent ankylosis (Figure 1).

**Table 1:** Heterotopic Ossification Classification System for Femoral Endoprostheses

Grade	Description
0	No HO
1	Bone island within soft tissue and >1 cm from the joint articulation or opposing bone surface
2	Bone spur from the bone-implant junction or diaphysis and >1 cm from the joint articulation or opposing bone surface
3	Bone spur or island <1 cm from the joint articulation or opposing bone surface
4	Apparent ankylosis of the joint

HO = heterotopic ossification.

Anterior-posterior and lateral radiographs from the most recent follow-up, at least 6 months postoperatively, were used to classify the pattern of HO using the defined classification system. For patients with a total femoral arthroplasty, the hip joint and knee joint were graded separately. Two PGY-5 orthopaedic surgery residents, graded the radiographs. The grading was repeated by the same two reviewers at a minimum of 4 weeks from the first review to determine intrarater reliability. Intra- and interrater reliabilities of the classification system were determined using the Cohen kappa coefficient (κ).

Four cases (9.8%) had a discrepancy in grade between the two initial graders, a consensus grade was determined by the senior author, an orthopaedic oncology fellowship-trained surgeon. This consensus grade was used for the final analysis. Final grades were used to determine the overall incidence and severity of HO in the cohort. Grading was stratified by the type of implant and by location (hip vs. knee). A chi squared test was used for statistical comparison of the incidence of HO and implant location. The incidence of HO also correlated with patient factors, including sex, age, Body Mass Index (BMI), diagnosis, comorbidities, and whether radiation was used postoperatively, using a chi squared test or Fisher exact test for categorical variables and an independent *t*-test for continuous variables. Before analysis, data were checked for normality. All analyses were done in SPSS (Version 26, IBM). Results were considered notable at *P* < 0.05.

## Results

Forty-one femoral endoprostheses in 41 patients were included in the study (22 proximal, 15 distal, and four total femur arthroplasties). The average patient age at surgery was 53 ± 23 years (range 9 to 91 years). Twenty-two patients were female (54%), and 19 patients were male (46%). The mean BMI at time of surgery was 30.0 ± 8.0 kg/m^2^ (range 15.6 to 61.6 kg/m^2^). Specific indications for femoral endoprosthetic reconstruction varied. In 85% (n = 35) of cases, the indication was due to cancer requiring bony resection with subsequent reconstruction. Fifteen percent (n = 16) were for revision arthroplasty with notable bone loss requiring endoprosthetic reconstruction. No patient received indomethacin or radiation for HO prophylaxis, although 14 patients (34.1%) received delayed postoperative radiation for cancer treatment, at least 14 days postoperatively after wounds were healed. The average time between surgery and final included postoperative radiographs was approximately 1 year (12 ± 7 months, range 6 to 34 months). No radiographs were included for analysis at <6 months to ensure that HO was mature and visible radiographically.

**Figure 2:**
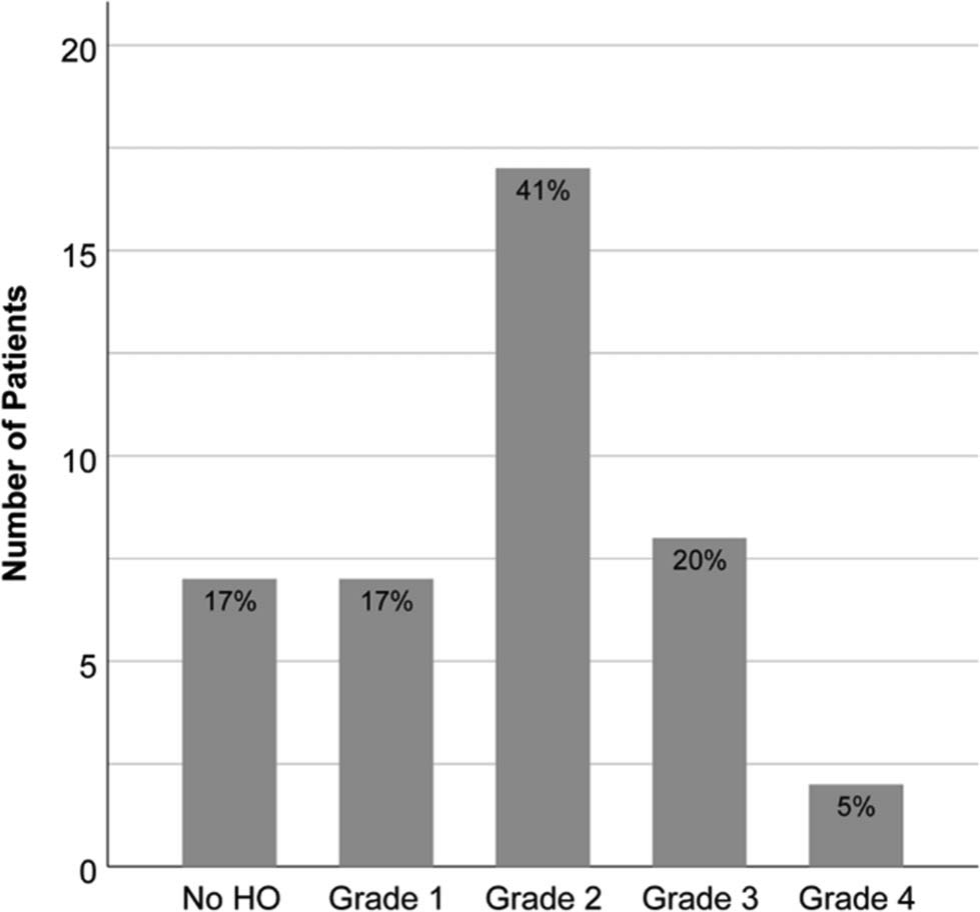
Bar graph showing number of patients and the percent within the entire cohort exhibiting each classification of HO. HO = heterotopic ossification.

Inter- and intrarater agreement was 87% (κ = 0.82) and 91% (κ = 0.88), respectively. The final HO classification grading data are summarized in Table 2. In total, an 83% incidence of HO was found after femoral endoprosthetic arthroplasty. In patients with a total femoral arthroplasty, two had HO at both joints, and two had no HO at either joint. Most patients exhibited grade 2 HO (Figure 2). All proximal femur arthroplasties, and 66.7% of distal femoral arthroplasties exhibited some degree of HO. Grade 4 HO was seen only in proximal femur arthroplasties, with an overall incidence of 5%. Implant location was a notable factor in whether the patient developed HO (*P* = 0.009, Table 3).

**Table 2:** Frequency of Heterotopic Ossification Grade Within Each Implant Location

Factor	Proximal (n = 22)	Distal (n = 15)	Total (hip) (n = 4)	Total (knee) (n = 4)	Total Cohort
No HO	0%	33% (5)	50% (2)	50% (2)	7 (17%)
Grade 1	18% (4)	20% (3)	0%	25% (1)	8 (20%)
Grade 2	46% (10)	47% (7)	0%	0%	17 (41%)
Grade 3	27% (6)	0%	50% (2)	25% (1)	8 (20%)
Grade 4	9% (2)	0%	0%	0%	2 (5%)

HO = heterotopic ossification.

**Table 3:** The Association of Heterotopic Ossification With Patient and Surgical Factors

Factor	Heterotopic Ossification		*P* Value
	No	Yes	
Implant location, n (%)			0.009
Proximal	0 (0)	22 (100)	
Distal	5 (33)	10 (67)	
Total (at the hip)	2 (50)	2 (50)	
Total (at the knee)	2 (50)	2 (50)	
Age, mean ± SD	30 ± 19	57 ± 21	0.003
BMI, mean ± SD	25.7 ± 4.9	30.8 ± 8.7	0.146
Sex, n (%)			1.0
Female	4 (18)	18 (82)	
Male	3 (16)	16 (84)	
Smoker (current), n (%)			0.075
No	7 (26)	20 (74)	
Yes	0 (0)	14 (100)	
Diabetes mellitus, n (%)			0.567
No	7 (19)	29 (81)	
Yes	0 (0)	5 (100)	
Indication, n (%)			1.0
Revision total joint	1 (17)	5 (83)	
Cancer	6 (17)	29 (83)	
Radiation, n (%)			0.075
None	7 (26)	20 (74)	
Cancer treatment	0 (0)	14 (100)	
Component failure, n (%)			1.0
No	5 (17)	24 (83)	
Yes	2 (17)	10 (83)	

Patients with HO were markedly older than those without (57 ± 21 vs. 30 ± 19; *P* = 0.003, Table 3). Sex, BMI, whether the patient was a smoker, and the diagnosis of diabetes did not demonstrate a notable association with HO (*P* = 1.0, *P* = 0.146, *P* = 0.075, *P* = 0.567). An indication of cancer did not affect incidence of HO (*P* = 1.0). Patients who underwent delayed postoperative radiation, at least 14 days postoperatively, and not within established window of 72 hours for postoperative HO prophylaxis, for cancer treatment also displayed no notable association with the presence of HO (*P* = 0.075). This is not surprising because prior studies indicate that for radiation to be effective for HO prophylaxis, it should be given within 72 hours of surgery.^1^ Overall, 12 patients (29%) in the entire cohort had a postoperative component failure. The presence of HO postoperatively did not reveal a notable impact on whether the component failed (*P* = 1.0).

## Discussion

Although several studies observe and report the incidence of HO following THA and TKA, to our knowledge, this study is the first to characterize HO around femoral endoprostheses.^3,7,10,16-18^ We found that HO is very common after femoral endoprosthetic reconstruction, with an overall incidence of 83% in our cohort, validating our hypothesis. The incidence of HO around the hip and proximal femur was greater than that around the knee and distal femur. All patients with a proximal femoral arthroplasty developed some degree of HO, whereas two-thirds of those with distal femoral arthroplasties exhibited HO. In addition, Grade 4 HO, representing apparent joint ankylosis, was only seen after proximal femur arthroplasties. This was not unexpected because HO is more common after THA than TKA.^1,3,16^

Symptomatic HO after THA and TKA remains uncommon, affecting only 0.9% to 10% of THA and <1% of TKA.^7,8,19,20^ Similarly, in this study, most HO that developed after femoral endoprosthetic reconstruction was low grade and distant from the joint articulation or opposing bone surface. Of those patients who developed HO, the majority were grade 1 or 2. This may suggest that most HO after femoral endoprosthetic reconstruction has lower potential clinical consequences, such as impingement, limited range of motion, or decreased functional outcome. Moreover, we found no association of the development of HO with component failure. Nevertheless, further studies would be needed to identify the clinical implications that HO has on functional outcomes after femoral endoprosthetic reconstruction and whether worse outcomes correlated with increasing HO grade.

The only notable patient factor associated with HO in our cohort was age. Patients who developed HO were older than those who did not (mean of 57 vs. 30). Sex, BMI smoking, and diabetes, some of which have been previously reported as risk factors for HO, did not show a notable impact in our cohort.^7,10^ However, because of the high incidence of HO within the cohort, and therefore few patients without HO for comparison, these analyses were likely not powered enough to detect a difference. We also looked at the impact of delayed radiation treatment, after wound healing, for oncologic treatment, because radiation can be used as prophylaxis for HO.^1,4^ We did not find a notable impact; in fact, all patients with cancer-related radiation developed postoperative HO. This would be expected because it has been shown that to be effective for HO prophylaxis, radiation must be given within 72 hours of surgery.^1^

No classification system has been described and validated for HO after femoral endoprosthetic reconstruction. The most standard classification for assessing HO is the Brooker classification, which describes HO after THA, and has been subsequently modified by Wright et al and Della Valle et al^13,17,21^ The Brooker classification System has also been modified to describe hip HO outside the setting of THA and for HO in other joints.^14^ In this study, we modified the Brooker classification system to create a novel classification for HO after femoral endoprosthetic reconstruction to account for the extensive bone resection. Compared with standard THA and TKA, femoral endoprosthetic reconstruction involves removal of at least a portion of the femoral diaphysis. This results in an increased distance between the remaining opposing bone surfaces (pelvis and femur, or tibia and femur). Therefore, in our classification, we included not only the distance between the HO to the opposing bone surface but also to the joint articulation surface. Our classification system demonstrated excellent intraobserver (κ = 0.88) and interobserver (κ = 0.82) reliabilities. These intra- and interobserver reliabilities are higher than those reported for the Brooker classification (0.74 and 0.43, respectively) and the Della Valle modification (0.78 and 0.59, respectively).^13,17^ The higher reliability values seen with the proposed classification may be attributable to the ease of identifying HO around the radiopaque prosthesis, particularly around the intertrochanteric region and the implant-diaphysis junction.

The limitations of this study must also be considered. First, this is a retrospective study that relies on the accuracy of the collected data and does not have standardized follow-up. Second, the study size is relatively small due to the lower frequency with which this procedure is performed. In addition, because the large majority of patients developed HO, the study is not highly powered for assessing risk factors. However, to our knowledge, no information about HO following implantation of a femoral endoprosthesis is currently published. Finally, clinical outcomes such as range of motion and patient satisfaction were not available. Further studies are needed to evaluate the clinical implications of the development of HO in this setting.

In conclusion, the proposed classification system has high reproducibility and agreement. Our data suggest that HO is common after femoral endoprosthetic reconstruction and is more predominant around the hip compared with the knee. Most HO was around the diaphyseal stem of the prosthesis and away from the joint, potentially having less of an effect on joint range of motion or function.
